# Intraocular pressure rise is predictive of vision improvement after intravitreal triamcinolone acetonide for diabetic macular oedema: a retrospective analysis of data from a randomised controlled trial

**DOI:** 10.1186/1471-2415-14-123

**Published:** 2014-10-21

**Authors:** Roderick F O’Day, Daniel Barthelmes, Meidong Zhu, Tien Y Wong, Ian L McAllister, Jennifer J Arnold, Mark C Gillies

**Affiliations:** Clinical Ophthalmology & Eye Health, The University of Sydney, Sydney, Australia; Singapore Eye Research Institute, National University of Singapore, Singapore, Singapore; Centre for Eye Research Australia, University of Melbourne, Melbourne, Australia; Lions Eye Institute, University of Western Australia, Perth, Australia; Marsden Eye Specialists, Sydney, Australia

**Keywords:** Intravitreal triamcinolone, Diabetic macular oedema, Vision improvement, Intraocular pressure rise, Adverse events, Efficacy

## Abstract

**Background:**

Intravitreal triamcinolone acetonide (IVTA) is an effective treatment for recalcitrant diabetic macular oedema (DMO). It has been shown to improve vision with benefits persisting up to five years. The most common initial side effect of IVTA treatment is rise in intraocular pressure, occurring in approximately 50% of patients within the first 6 months of treatment. We evaluated whether there is a correlation between the development of intraocular pressure rise and improvement in vision.

**Methods:**

Analysis of individual data from 33 eyes of 33 participants treated with IVTA for DMO from a prospective, randomised, double-masked, placebo controlled trial. The degree of intraocular pressure (IOP) rise was correlated with improvement in best-corrected visual acuity (BCVA) at 1 and 6 months.

**Results:**

The proportion of eyes gaining 5 or more logMAR letters was higher in eyes with greater IOP rise (*p* = 0.044). Better absolute improvement in BCVA at 6 months (*p* = 0.045) was also found in eyes with greater IOP rise. Regression analyses revealed a correlation between IOP rise of 10 or more mmHg and absolute BCVA improvement at 6 months (odds ratio 1.22, 95% confidence interval 1.01-1.48, *p* = 0.039), but not at 1 month.

**Conclusions:**

IOP rise and vision improvement appear to be correlated following IVTA for DMO, suggesting that the mechanisms that cause both may be linked.

**Trial Registration:**

Clinical trials.gov NCT00167518, September 5, 2005.

## Background

The treatment of diabetic macular oedema (DMO), the commonest cause of vision loss in people with diabetes, is rapidly evolving [1]. The past ten years have seen a progressive shift away from laser photocoagulation therapy with the emergence of new pharmacotherapeutic approaches. Much of the recent focus has been on intravitreal inhibitors of vascular endothelial growth factor (VEGF). These are rapidly becoming the standard of care for diffuse DMO [2]. VEGF inhibitors are generally well tolerated, however, potentially significant systemic and ocular side effects have not been comprehensively excluded [3, 4]. In particular, it has been suggested that VEGF may play a neuroprotective role, the inhibition of which may lead to loss of vision in the long-term [5].

Intravitreal triamcinolone (IVTA) is another proven treatment for recalcitrant DMO with well-known side-effects [6–12]. In patients with persistent DMO despite laser treatment, intravitreal injections of triamcinolone acetonide were shown to improve vision and reduce macular thickness with benefits persisting up to five years [6–8]. In that study, IVTA treatment doubled the chance of visual acuity improvement and halved the chance of deterioration in eyes with advanced DMO [6–8].

The ocular safety of IVTA treatment has been thoroughly explored [6–8, 13–16]. The most common adverse events of IVTA treatment are intraocular pressure (IOP) rise and accelerated cataract formation, particularly posterior subcapsular cataract. Reported rates of IOP rise differ; in our study approximately 50% of eyes had an IOP rise of 5 or more mmHg within the first six months of IVTA treatment [6–8]. IOP rise has been shown to be significantly associated with cataract progression, suggesting that these steroid-related adverse events may have similar aetiologies, for example genetic polymorphisms in the steroid receptor [15]. In this study, we analysed whether IOP rise was linked with visual acuity improvement using data from the Triamcinolone for Diabetic Macular Oedema (TDMO) study [6–8]. If so, this would suggest that the mechanisms of IOP rise and vision improvement after IVTA for DMO are similar and may help to further advance our understanding of how IVTA acts on macular oedema.

## Methods

This analysis is based on data from the TDMO study, which was the first major prospective, randomised, double masked, placebo-controlled clinical trial to test the hypothesis that IVTA safely improves visual acuity in eyes with advanced DMO over 2 years [6–8]. For the TDMO study, patients were recruited from a major tertiary referral centre. Inclusion criteria were persistent diabetic macular oedema involving the central fovea persisting three months or more after adequate laser treatment and best corrected visual acuity (BCVA) in the affected eye of 6/9 or worse [6–8]. A total of 69 eyes were enrolled in the study, 4 of which were lost to follow up within the first three months. Of the remaining 65 eyes, 33 received triamcinolone treatment and are the subject of the present analysis. At baseline eyes received either a 4 mg IVTA injection – 0.1 ml of Kenacort 40 (40 mg/ml triamcinolone acetonide; Bristol-Myers Squibb, Noble Park, Australia) – or a sham-injection with subconjunctival saline. The TDMO study was conducted in accordance with the Declaration of Helsinki and was approved by the South Eastern Sydney Area Health Service and University of Sydney research ethics committees. Written informed consent was obtained from all patients prior to treatment assignment.

For the purpose of this analysis, the visual acuity outcomes were (A) change in number of letters read on a Logarithm of the Minimal Angle of Resolution (LogMAR) chart and (B) proportion of eyes that had improved vision by 5 or more LogMAR letters. Both were assessed as difference between baseline and 1 or 6 months. During this time all eyes received a single injection of IVTA. Change in IOP was defined as the difference between baseline and the highest IOP measured within 6 months after IVTA treatment. IOP was measured using Goldmann applanation tonometry. Various definitions of IOP rise are used in the literature including, absolute values [17, 18] percentage increase from baseline [19], and change from baseline [13–19]. No one definition has been universally adopted. For a descriptive analysis, we stratified eyes into three groups according to degree of change in IOP – less than a 5 mmHg increase, increase by 5 mmHg or more but less than 10 mmHg and increase by 10 mmHg or more – from baseline. Intraocular pressure and VA data at 1 and 6 months were compared to baseline. This enabled us to stratify our analyses to effectively assess eyes that have a moderate intraocular pressure response, i.e. those gaining 5 or more, but less than 10 mmHg, and those that have a strong intraocular pressure response, i.e. those gaining 10 or more mmHg. For the regression analysis, eyes were allocated to two groups: less than 10 mmHg in increase and 10 mmHg or more increase.

### Statistical analysis

Data are presented as mean ± standard deviation (SD) when normally distributed or as median and [interquartile range] if not. Normality was assessed using the Shapiro-Wilks test. The homogeneity of variances between two groups was assessed using Levene’s test. Differences in continuous variables between multiple groups were compared using a one-way ANOVA or the Kruskal-Wallis for normally and not normally distributed data, respectively. In cases of significant ANOVA results, post-hoc testing was performed. If equal variances could be assumed, a Bonferroni correction was applied. If not, Dunn’s test was applied. Differences in proportions between three groups were analysed using Fisher’s exact test with the Freeman-Halton extension. Correcting for baseline characteristics, two separate binary logistic regression models were performed to examine whether improvement in vision at 1 (model A) and 6 months (model B) was predictive of IOP increase. Eyes with missing values were excluded from these analyses. The dependent variable was IOP rise within six months of IVTA injection. The independent variables were change in BCVA (continuous variable) at 1 or 6 months, age at baseline (continuous variable), gender (binary variable) and phakic status at baseline (binary variable). Collinearity was examined by calculating tolerance and variance inflation factor (VIF).

*A priori* a *p* value of <0.05 was defined as statistically significant. All data were analysed using a commercially available software package (PASW 21, SPSS Inc., Chicago, IL, USA).

## Results

Of the 33 eyes treated with IVTA in the TDMO study, 6 (18%) were pseudophakic at baseline and 15 (45%) were female. The mean age of patients at baseline was 63.27 ± 10.10 years and median glycosylated haemoglobin (HbA1c) was 7.7 [5.7 to 9.5] percent. The mean central macular thickness (CMT) was 444 ± 125 micrometres, IOP was 16.55 ± 2.56 mmHg and median BCVA was 64.0 [52.5 to 70.0] letters logMar.

Table 1 shows the baseline characteristics of eyes treated with IVTA stratified with respect to intraocular pressure response. There was no significant difference in the measured baseline characteristics – age, gender, HbA1c, phakic status, IOP, BCVA and CMT – between the groups classified according to degree of IOP rise.

**Table 1:** **Baseline characteristics of 33 eyes treated with IVTA stratified with respect of change in IOP from baseline**

Characteristic	IOP change < 5 mmHg rise	IOP rise 5–9 mmHg	IOP rise ≥ 10 mmHg	***P value***
	(n = 17)	(n = 8)	(n = 8)	
**Age (y)**	63.8 ± 8.4	63.9 ± 15.7	61.5 ± 7.2	0.864
**Gender, female (n)**	9 (53%)	4 (50%)	2 (25%)	0.440
**HbA1c** ^**a**^	7.9 ± 1.2	9.1 ± 1.5	8.2 ± 1.5	0.295
**Phakia, pseudophakic (n)**	3 (18%)	2 (25%)	1 (14%)	0.862
**IOP (mmHg)**	16.9 ± 2.4	15.8 ± 3.1	16.6 ± 2.6	0.600
**BCVA (letters)**	60.1 ± 14.0	62.9 ± 6.7	58.5 ± 12.7	0.771
**CMT (μm)** ^**b**^	436 ± 146	443 ± 132	468 ± 22	0.916

IVTA = intravitreal triamcinolone; IOP = intraocular pressure; BCVA = best-corrected visual acuity; CMT = central macular thickness.

Values are mean ± standard deviation or median with interquartile range shown in box brackets.

^a^n = 13, 4 and 7 for IOP rise <5 mmHg, 5-9 mmHg and ≥10 mmHg, respectively.

^b^n = 12, 5 and 4 for IOP rise <5 mmHg, 5-9 mmHg and ≥10 mmHg, respectively.

Data on IOP rise was available for all eyes. BCVA data was not available for 4 eyes at the 6 month follow-up, but was otherwise complete.

### IOP rise and visual outcomes

Seventeen eyes (52%) had an IOP change from baseline of less than a 5 mmHg or more increase, 8 (24%) had a rise of 5 or more but less than 10 mmHg and 8 (24%) had a rise of 10 or more mmHg. Eyes that had greater IOP rise had significantly greater chance of having an improvement in vision by 5 or more letters at 1 month (*p* = 0.044, Table 2) and there was a trend for these eyes to have better absolute improvement in BCVA (*p* = 0.058, Table 2). At 6 months, eyes that exhibited greater IOP rise had better absolute improvement in BCVA (*p* = 0.045, Table 2), but the proportion of eyes improving by 5 of more letters was not significant (Table 2).

**Table 2:** **Vision improvement in eyes treated with IVTA stratified with respect to change in IOP from baseline**

	IOP change < 5 mmHg rise	IOP rise 5–9 mmHg	IOP rise ≥ 10 mmHg	***P value***
	(n = 17)	(n = 8)	(n = 8)	
**1 month**				
Gain in BCVA ≥ 5 letters	8 (47%)	2 (25%)	7 (88%)	0.044
Mean gain in visual acuity (letters)	4.0 [-0.5 to 6.0]	3.0 [0.0 to 6.3]	6.5 [5.3 to 12.0]	0.058
**6 months**				
Gain in BCVA ≥ 5 letters^a^	9 (56%)	3 (43%)	5 (71%)	0.561
Mean gain in visual acuity (letters)^a^	4.8 ± 6.5	1.4 ± 7.4	11.7 ± 9.7	0.045

IVTA = intravitreal triamcinolone; IOP = intraocular pressure.

Values are mean ± standard deviation or median with interquartile range shown in box brackets.

^a^n = 16, 7 and 7 for IOP rise <5 mmHg, 5-9 mmHg and ≥10 mmHg, respectively.

Regression analyses revealed a correlation between IOP rise of 10 or more mmHg and absolute vision improvement at 6 months (odds ratio 1.22, 95% confidence interval 1.01-1.48, *p* = 0.039, Table 3), but not at 1 month. Baseline age, gender and phakic status did not predict IOP rise by 10 or more mmHg (Table 3).

**Table 3:** **Predicting IOP rise of 10 or more mmHG**

Predictor	Odds ratio	95% confidence interval	***P value***
**1 Month**			
Baseline age	1.00	0.91 – 1.10	0.972
Gender	2.61	0.39 – 17.62	0.325
Baseline phakia	1.32	0.10 – 17.05	0.831
Mean gain in visual acuity at 1 month (letters)	1.13	0.96 – 1.33	0.149
**6 months**			
Baseline age	0.99	0.87 – 1.14	0.894
Gender	3.91	0.39 – 39.58	0.248
Baseline phakia	7.94	0.14 – 457.82	0.316
Mean gain in visual acuity at 6 months (letters)	1.22	1.01 – 1.48	0.039

IVTA = intravitreal triamcinolone; IOP = intraocular pressure; BCVA = best corrected visual acuity.

Binary logistic regression analyses.

## Discussion

This analysis examined the relationship between the development of adverse events and visual outcomes after IVTA treatment in order to gain further insights into its mechanism of action in treating DMO. We found a correlation between IOP rise and vision improvement at two time points in the first 6 months after a single treatment with IVTA for DMO. It seems that the greater the IOP rise, the more likely eyes are to improve in vision, at least in the short term.

Significant research has examined the clinical application of glucocorticoids in inflammatory and oedematous diseases of the eye, however, the mechanisms of steroid-induced changes are yet to be fully elucidated. Steroid-induced IOP rise is thought to be due to activation of glucocorticoid receptors in the trabecular meshwork [20, 21]. It has been suggested that polymorphisms of the Myocilin gene, which is upregulated by glucocorticoids, may be responsible for steroid-induced IOP rise [22]. Ultimately, steroids cause biochemical and ultrastructural changes to the trabecular meshwork resulting in greater resistance in the aqueous humour outflow tract [23, 24]. Less is known about the mechanism of vision improvement by IVTA than that of IOP rise. It is likely to be more complex than reduction in macular thickness alone, as evidenced by a lack of a correlation between these two clinical outcomes [25]. It has been proposed that improvement in glial and neuronal function may explain the improvement in vision following IVTA treatment [26–28]. This is consistent with recent clinical findings that there is a strong link between photoreceptor integrity and visual acuity outcomes in patients treated with IVTA for DMO [29].

A strong correlation between IOP rise and cataract formation has previously been shown, suggesting a common mechanism to their formation, possibly through genetic polymorphisms of the common steroid receptor in the trabecular meshwork and lens epithelium [15, 20, 21, 30]. We conducted the present analysis to see whether there was a similar correlation between IOP rise and visual outcomes after intraocular steroid therapy. Our results suggest that this is the case and that there may be a common mechanism for IOP rise and vision improvement. The correlation found in this analysis was not as strong as that between IOP rise and cataract formation [15]. This may be due to the small numbers available for analysis. Despite this, there was a statistically significant trend for eyes that had greater IOP rise to have good visual outcomes and the study that the data is derived from is of very high quality. The fact that the association between IOP rise and VA improvement could only be established for the 6 month VA outcomes may be related to the fact that in DMO, VA improvements occur slowly, especially in the patient group chosen for this study – patients with recalcitrant DMO.

The major weakness of this retrospective analysis is the relatively small number of patients available. In theory, this would result in missing weak relationships. Our findings should be viewed positively in light number of patients analysed. The classifications of IOP rise we used were not prospectively defined. Analyses based on these are potentially subject to selection bias. None of the measured baseline characteristics were found to be statistically significantly different between the groups. We cannot be certain that unmeasured baseline characteristic influenced our results.

As well as potentially adding to our knowledge of the mechanisms of IVTA on macular oedema, this analysis has clinical implications. It seems that those eyes that have a high degree of IOP rise are more likely to have very good visual outcomes 6 months after treatment, with a mean improvement of 11.7 letters on an ETDRS scale found in our subjects. Concurrently, these eyes are at greater risk of developing posterior subcapsular cataract [15]. This adds further complexity to the harm/benefit analysis the clinician must make in deciding whether to treat.

## Conclusions

This analysis has revealed a positive relationship between the intraocular pressure rise and visual improvement following IVTA treatment for DMO, at least in the short term. This suggests that there may be a common mechanism to the development of these outcomes, which is yet to be elucidated. These eyes are concurrently at higher risk of developing posterior subcapsular cataract.
